# Continuous Intraoperative Nerve Monitoring in Thyroid Surgery: Can Amplitude Be a Standardized Parameter?

**DOI:** 10.3389/fendo.2021.714699

**Published:** 2021-08-03

**Authors:** Sara Mazzone, Adelaide Esposito, Vittorio Giacomarra

**Affiliations:** ENT Department, Santa Maria degli Angeli General Hospital, Pordenone, Italy

**Keywords:** thyroid surgery, continuous nerve monitoring, vocal cord paralysis, vagus nerve intraoperative monitoring, thyroid cancer

## Abstract

The objective of this study is to evaluate electromyographic waveforms related to vagus monitoring. We collected data from patients undergoing thyroidectomy with CIONM, regardless of vocal cord response amplitude initially measured. We divided data of 193 nerves into three groups, according to initial amplitude value: ≥500 µV (Group 1,110 pt.), between 100 and 500 µV (Group 2, 79 pt.), and <100 µV (Group 3, 4 pt.). ROC curve showed a high diagnostic accuracy of final amplitude absolute value in vocal cord paralysis detection in both groups (89 and 86%). An increase of vocal cord paralysis risk was associated with progressive amplitude reduction (Group 1: OR=1.05, CI=1.02–1.09, p=0.001; Group 2: OR=1.05, CI=1.02–1.08, p=0.002). Cut-off values for amplitude reduction with optimal sensitivity and specificity were −77% in Group 1 and −15% in Group 2. In Group 3 signals showed an amplitude <100 µV for all monitoring, with no loss of a recognizable signal and normal postoperative cordal functionality. The use of a strict amplitude signal cut-off value ≥500 µV could be too restrictive. Also, signal with baseline amplitude <500 µV may be considered equally adequate. Setting the alarm for a reduction of 77% in patients with initial amplitude ≥500 µV and of 15% for those <500 µV could make monitoring safe and an effective aid for surgeons. In conclusion, there are cases in which initial amplitude is lower than that considered as adequate by current literature but with well recognizable and stable EMG waveforms. How those cases should be approached and what should the surgeon’s attitude be are a matter of discussion.

## Introduction

Vocal cord paralysis (VCP) is the main complication of thyroidectomy, representing a life-threatening event in case of bilateral damage.

VCP incidence varies greatly among different studies with an incidence ranging from 2.3 to 26% for transient forms and from 0.3 to 6% for permanent forms, with values increasing up to 14% in cases of revision surgery (1–3).

Even if visual nerve identification represents the gold standard to prevent nerve injury, intraoperative neuromonitoring (IONM) can be considered as an additional technique for intraoperative nerve management and postoperative vocal cord function prognostication.

The use of IONM is recommended in all cases of total thyroidectomy, but especially in cases of thyroid cancer, revision thyroid surgery, or preoperative vocal cord dysfunction (4).

Continuous IONM (CIONM) allows real-time monitoring of nerve functional state, being able to recognize any potential nerve damage and to guide the surgeon in sudden corrective maneuvers aimed to preserve the functional nerve integrity.

Considering the progressive worldwide diffusion of the technique, the International Neural Monitoring Study Group (INMSG) issued state-of-the-art guidelines to uniform indication, technique, and electromyographic (EMG) waveform reading (5).

In this study we evaluated EMG waveforms related to the monitoring of 195 nerves, with particular focus on the amplitude of the recorded signals. Our evaluation led to some important issues that could have an impact on both signal interpretation and clinical practice.

## Material and Methods

In this prospective single-institution study, we collected data from patients undergoing thyroid surgery with continuous intraoperative vagus nerve monitoring.

No case selection was conducted, and surgical indication was in accordance with International Guidelines (6, 7).All patients were informed about the use of CIONM and signed an informed consent.

Each patient underwent pre- and postoperative fiberoptic laryngoscopy; patients with preoperative cord paralysis were excluded from the study.

For each patient was used a short half-life non-depolarizing muscle relaxant (rocuronium at 0,5 mg/kg). Train of four (TOF) stimulation was used for intraoperative neuromuscular transmission monitoring, considering the TOF % as a reference parameter (ratio between the fourth and the first muscle response).

Vocal cord muscle depolarization detection was achieved by using an adhesive tube electrode (Dr. Langer Medical GmbH) placed 7–10 mm above the upper edge of the cuff; the electrode was wrapped around the endotracheal tube, covering the external lumen at 360° and allowing to get thoroughly in contact with the vocal cords. Tube-positioning check was performed either by direct or video laryngoscopy, by evaluation of respiratory variation of the baseline, and by a so-called “tap test,” even though it is an approximate method providing no quantitative data (8).

Surgery was made according to classic technique; we identified the vagus nerve in the neurovascular bundle, and before positioning the vagal probe, we checked neuromuscular circuit integrity by manual probe; visual identification of recurrent laryngeal nerve (RLN) was performed in all cases. Probe placement time and nerve monitoring did not significantly affect overall surgical time.

CIONM was used in all patients, regardless of vocal cord response amplitude initially measured, using Dr. Langer Medical GmbH’s IONM system AVALANCHE® SI. This device offers seamless recurrent nerve monitoring *via* continuous vagus nerve stimulation (Saxophone electrode, Dr. Langer Medical GmbH), as well as the possibility of functional integrity check of motor nerves *via* intermittent stimulation by using a handheld stimulation probe (disposable monopolar probe, Dr. Langer Medical GmbH).

Stimulation is performed with a stimulus duration of 200 µs and by a current of 1–2 mA, with 4 stimuli per second in intermittent stimulation and with 3 stimuli per second during continuous one.

Stimulation triggered MUAPs (Motor Unit Action Potential Duration) are filtered and amplified analogically and digitally. These high-resolution signals are presented to the end-user as a graphic and audible signal. Further to signal detection, this kind of signal processing offers the possibility to detect smallest signal changes immediately. The device automatically stores all signals in a database, allowing for a full postoperative signal review.

In our Institute this instrumental method is routinely used, and data analysis has not led any variation of patients’ management intra- and postoperatively. Thereby, considering study characteristics, patients’ enrollment, collection, and data analysis, for this study approval of ethics committee does not apply.

Dr. Langer Medical GmbH provides two stand-alone software tools: the “PC Viewer” application allows postoperative signal review, while the second tool allows the user to review all the stimulation data (time and date, stimulation current, amplitude, latency, stimulation probe used) in an MS Excel file.

Continuous variables were expressed as mean (M) and standard deviation (SD), categorical variables as number (N) and percentage (%). The Student 2-sample *t* test and Pearson χ2 test were used to compare the differences between two groups for continuous and categorical variables, respectively.

Predictors of VCP were evaluated with logistic regression analysis, expressed as odds ratio, and 95% confidence interval for continuous variables and with Cochran and Mantel-Haenszel odds ratio for categorical variables. Multivariate models were performed with logistic regression analysis to create adjustment for age, gender, type of thyroid disease, malignancy, and side of lesion.

A Receiving Operating Characteristic (ROC) curve analysis, which reported the area under the curve (AUC) with a 95% confidence interval (CI), was performed to determine the diagnostic accuracy for VCP prediction and cut-off values; optimal sensitivity and specificity were calculated using Youden index. Significance was defined as a p value <0.05. All statistics were performed with SPSS version 26 (IBM Corporation).

## Results

Over a 17-month period, from April 2018 to September 2019, we performed thyroid surgery with CIONM in 108 patients, 27 men (age range 31–79 years, mean 57,93 years) and 81 women (age range 19–82 years, mean 59,51 years). In 87 cases a total thyroidectomy and in 21 cases a loboistmectomy (14 right, 7 left) were performed, for a total of 195 dissected nerves (99 right, 96 left). Two nerves were excluded from the analysis: in one case, a worsening of a bradycardia already present at anesthetic induction occurred after placement of the vagal stimulation probe, which was removed, with restoring the previous values of heart rate; in another case we intraoperatively found a “non-recurrent” recurrent laryngeal nerve, with cranial emergence at the site of the probe positioning and non-valid vagal signal. We therefore analyzed data related to 193 nerves.

In 71 cases surgery was performed for benign pathology (Multinodular Goiter, Adenoma, Graves-Basedow’s disease, Thyroiditis). In 37 cases histology confirmed the malignant nature of the pathology: in 33 cases it was a papillary Ca., in 1 case it was follicular Ca., and in 3 cases it was a medullary Ca. In three cases a central compartment lymph node dissection was performed; 10 patients underwent a lateral compartment-oriented selective neck dissection, according to International Guidelines, since there was a neoplastic lymph node involvement at the time of diagnosis (7).

With the exclusion of the case of intraoperative bradycardia, no cardiopulmonary complications occurred. In three cases we experienced an endotracheal tube displacement in caudocranial direction and had to put it back into the right position, with amplitude response retrieval. In three cases we had to interrupt the surgical procedure and did not complete the thyroidectomy due to the absence of vagal signal at the end of first thyroid lobe removal; in all three cases we observed a transient vocal cord palsy.

In our group of 193 nerves, we observed 11 cases of transient vocal cord palsy (5,70%) and 4 cases of permanent palsy, in two of which there was a nerve neoplastic infiltration (2.07%).

We divided nerves into three groups, according to the amplitude value of the signal obtained at the placement of the vagal probe. In 110 cases the initial amplitude of vagal stimulation response was ≥500 µV (Group 1), and in 79 cases it was between 100 and 500 µV (Group 2). The Group 3 includes four nerves who had an initial amplitude value <100 µV (**Table 1**).

**Table 1:** Main features of EMG signals for Groups 1, 2, and 3. In Group 1 one nerve had A1=A2.

	Number of nerves	Mean initial amplitude [µV]	Mean final amplitude [µV]	Number of nerves with amplitude decrease	Number of nerves with amplitude increase	Mean amplitude decrease (%)	Mean amplitude increase (%)	Early postoperative VCP	Permanent VCP
Group 1	110	895	670	77	32	44,59%	36,85%	5	3
Group 2	79	306	322	43	36	34,42%	75,24%	6	1
Group 3	4	77	71	3	1	23,56%	70%	0	0

No statistical differences between Group 1 and Group 2 were found in age (Group 1: 59±14 years *vs* Group 2: 57±14 years; p=0.234), rate of malignancy (35 *vs* 37%; p=0,759), lesion size >80 cm^3^ (24 *vs* 20%; p=0,093), or side (left side 47 *vs* 57%; p=0.102); on the other hand, male gender was prevalent in Group 1 (80 *vs* 67%: p=0.044).

Group 3 was excluded from statistical analysis due to the limited sample size.

### Prediction of VCP by Final Amplitude Absolute Value in Group 1 and Group 2

ROC curve showed a high diagnostic accuracy of final amplitude absolute value in VCP detection in both Group 1 and Group 2, 89 and 86%, respectively. In particular, the area under the ROC curve for Group 1 was 0.890 (0.702–1.000; p<0.0001) and for Group 2 was 0.861 (0.702–1.000; p<0.002).

### Prediction of VCP by Amplitude Variation in Group 1 and Group 2

**Figure 1:**
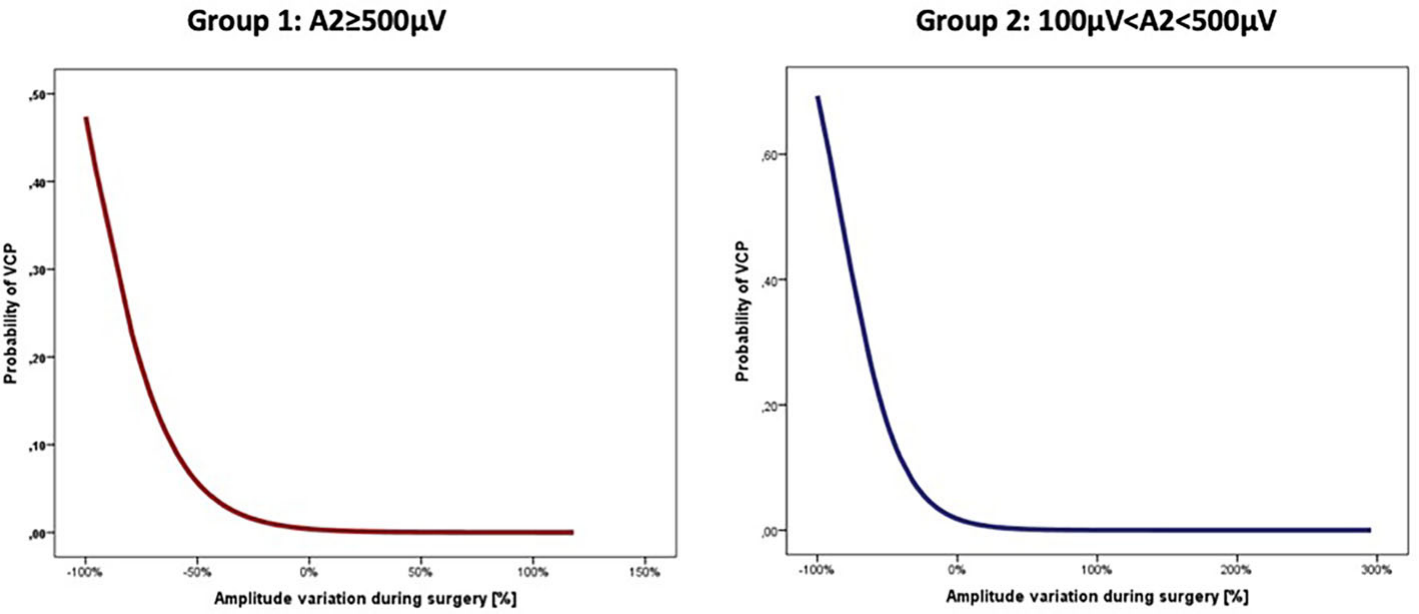
Linear correlation between probability of VCP and amplitude variation during surgery.

The data analysis of the two groups shows that an increase of VCP risk was associated with the progressive amplitude reduction during intervention in both groups (Group 1: OR=1.05, CI=1.02–1.09, p=0.001; Group 2: OR=1.05, CI=1.02–1.08, p=0.002), corresponding to a mean increase of 5% of the relative risk of VCP per each percentage value of amplitude decrease in both groups (**Figure 1**).

The association between amplitude reduction and VCP risk remained independent even after multivariate analysis including age, gender, disease, and side of lesion in both groups (Group 1: OR=1.05, CI=1.02–1.09, p=0.003; Group 2: OR=1.05, CI=1.01–1.09, p=0.005).

**Figure 2:**
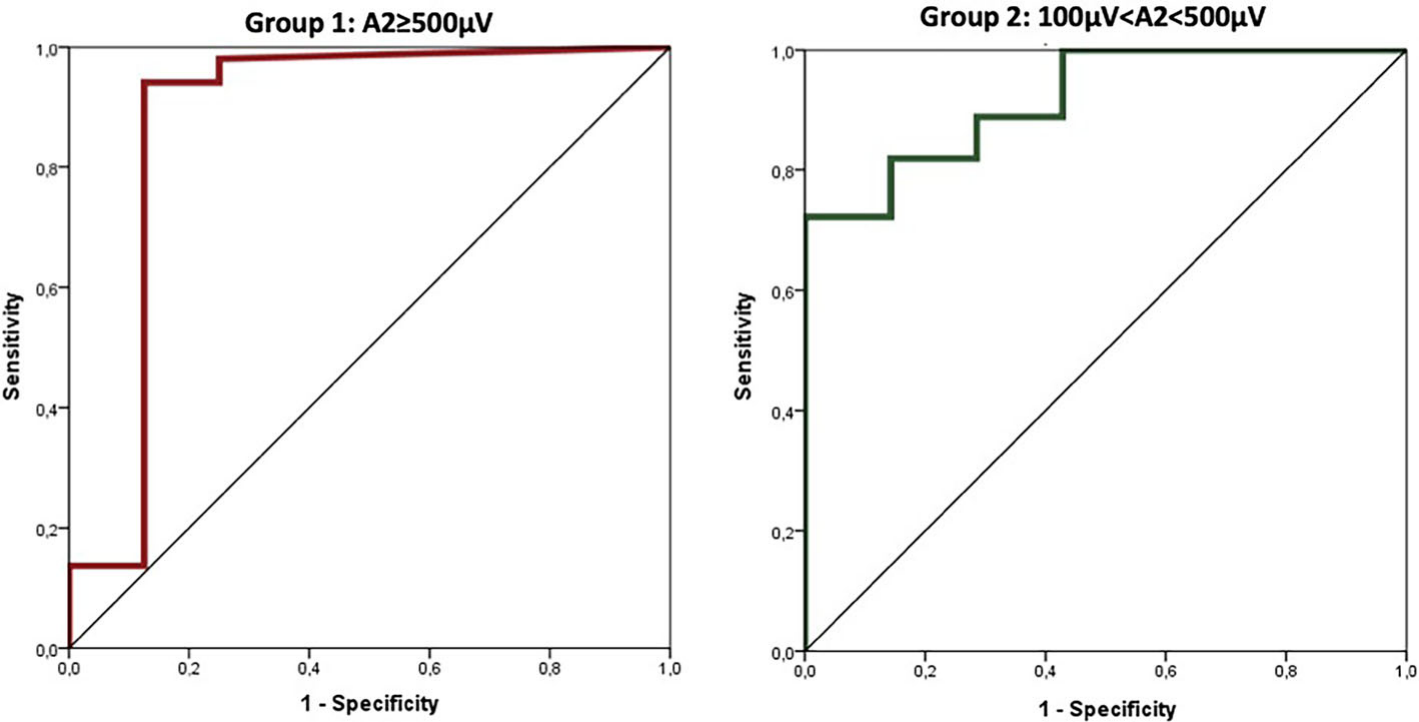
ROC curves analysis for diagnostic accuracy of amplitude reduction in VCP detection.

ROC curve showed a high diagnostic accuracy of amplitude reduction in VCP detection in both Group 1 and Group 2, 88 and 92%, respectively. In particular, the area under the ROC curve for Group 1 was 0.877 (0.682–1.000; p<0.0001) and for Group 2 was 0.919 (0.833–1.000; p<0.0001) (**Figure 2**).

**Figure 3:**
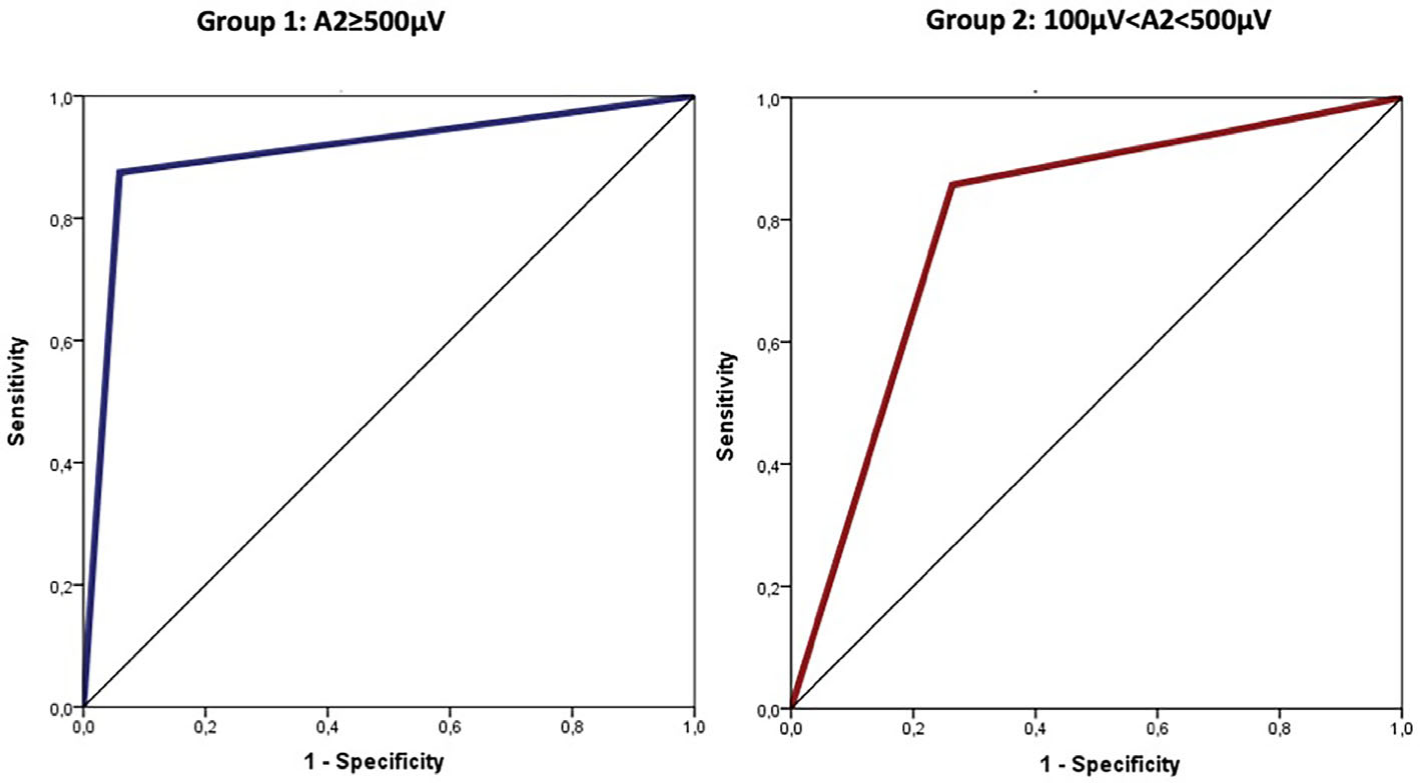
Cut-off values for amplitude reduction with optimal sensitivity and specificity were −77% in group 1 (≥500 µV) and −15% in group 2 (between 100 and 500 µV).

Using ROC curves to determine the diagnostic accuracy of VCP, cut-off values for amplitude reduction (calculated using Youden index) with optimal sensitivity and specificity were −77% in Group 1 (≥500 µV) and −15% in Group 2 (between 100 and 500 µV). The area under the ROC curve for amplitude reduction (Group 1, cut-off 77) was 0.908 (95%CI: 0.770–1.000; p<0.0001) corresponding to sensitivity=87% and specificity=94%. Similarly, the area under the ROC curve for amplitude reduction (Group 2, cut-off 15) was 0.797 (95%CI: 0.632–0.961; p=0.010) corresponding to sensitivity=86% and specificity=74% (**Figure 3**).

### Group 3

**Figure 4:**
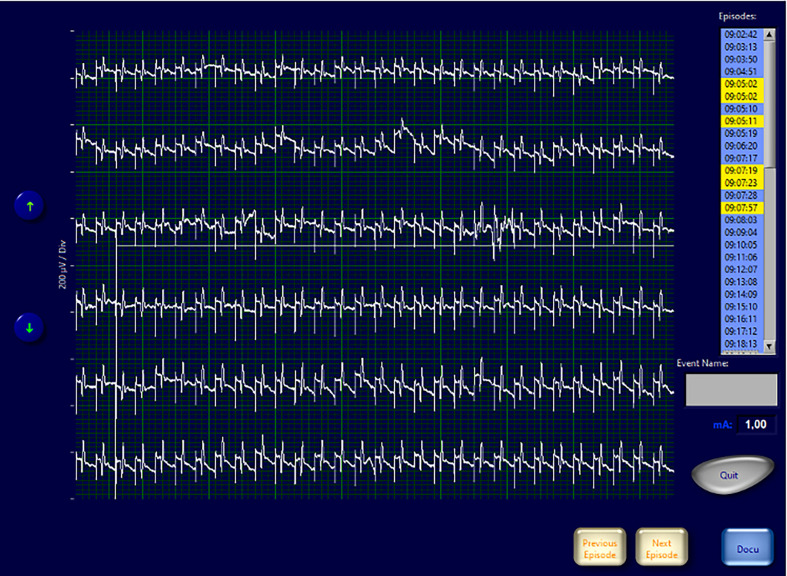
EMG frame that shows EMG waveforms during continuous vagal stimulation with amplitude <100 µV in a patient with normal pre- and postoperative vocal cord function.

**Figure 5:**
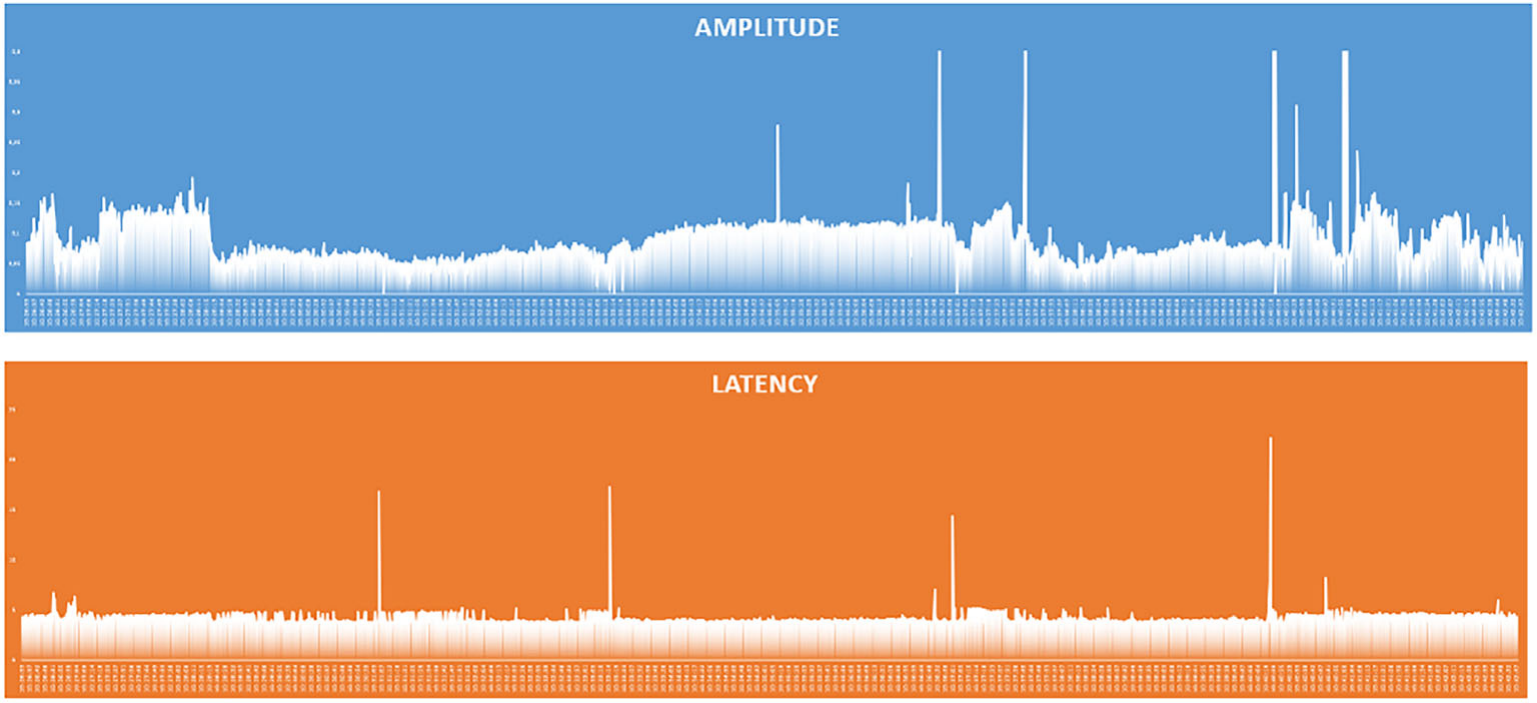
Graphical representation of amplitude and latency values of the responses of the continuous vagal stimulation in a patient with amplitude <100 µV and normal pre- and postoperative vocal cord function.

**Figure 6:**
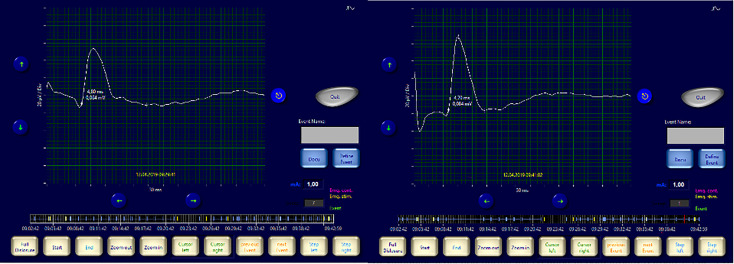
Right vagus nerve signal (Amplitude <100 µV): before and after lobectomy.

Interestingly in four cases, despite having ensured the correct positioning of endotracheal tube, the absence of neuromuscular blocking agents, and the functionality of the monitoring system, the EMG signals showed an amplitude <100 µV since the positioning of the probe and for the entire duration of the monitoring. In none of these cases we experienced loss of a recognizable signal with defined amplitude and latency and observed postoperative cordal paralysis. **Table 2** shows the main features of EMG signals for these nerves (**Table 2**). In **Figures 4**–**6** we show an explicative case in which the right vagus nerve shows an amplitude value lower than 100 µV for the whole surgery, despite a normal postoperative cordal motility.

**Table 2:** Main characteristics of waveform with amplitude <100 µV.

PATHOLOGY	A1 [µV]	A2 [µV]	Amplitude decrease [%]	Amplitude increase [%]	VCP	% single event: decrease amplitude of 50%	% single event: latency increase of 10%	Max consecutive time of both events (s)
Papillary ca	82	64	21,95	–	no	12,54	30,3	7
Benign	91	71	21,98	–	no	23,52	4,95	7
Benign	86	63	26,74	–	no	1,73	11,5	8
Benign	50	85	–	70,00	no	0	26,48	–

## Discussion

Visual identification of RLN still represents the gold standard for prevention of recurrential damages. However, nerve identification could be difficult due to several anatomical variables, particular clinical pictures, or revision surgery (9). Moreover, the nerve anatomical integrity may not correspond to the functional integrity. The authors agree that in most cases (75–83%) the RLN damage is caused by a traction injury, particularly in correspondence with the Berry’s ligament; other causes of nerve damage include thermal, compressive, clamping and ligature damage, entrapment, suction-related injury, and transection (10–12). A study by Dionigi et al. showed that in a group of 281 nerve injuries, only 14% of the damages would have been identified intraoperatively with direct visualization and without nerve functional monitoring (13). In the last years, IONM during thyroid surgery has gained worldwide acceptance as additional aid for visual RLN identification. According to literature, the main applications of IONM are identification of the RLN, aid in dissection, lesion site identification, and postoperative neural function prognostication (8). Although some reviewers consider the advantages linked to intermittent IONM as limited, especially for damage prevention, IONM is employed worldwide, as recommended by the American Head and Neck Society, especially in all thyroid cancer cases and particularly in those complicated by pre-surgery RNL paralysis and in revision surgeries (14).CIONM represents instead an important evolution that is progressively showing a superiority over intermittent monitoring in preventing vocal cord palsy (15). The periodic automatic electrical stimulation of the vagus nerve with an intensity of 1–2 mA causes the contraction of vocal cord muscles, whose activity is converted into an acoustic and visual signal; the surface electrode in contact with the vocal cords records the electromyographic waveform during the whole intraoperative stimulation. The real-time monitoring of the whole vagus-recurrent circuit during dissection allows immediate assessment of imminent damage and consequent corrective surgical maneuvers, thanks to early acknowledgment of variation in the electromyographic waveform. As known, the response signal to the vagal stimulus is represented by a biphasic wave corresponding to the action potential of the motor unit of the ipsilateral thyroarytenoid muscles. Each wave is defined by two parameters: the amplitude, which can vary significantly from subject to subject and is correlated with the number of muscle fibers that participate in the depolarization during stimulation, and the latency, which corresponds to the time elapsed from stimulation to the first peak of the muscle response. After probe positioning, a reference EMG response, defined as Baseline, is acquired. During the whole monitoring, the changes in EMG response are therefore analyzed and compared to baseline. Due to relatively recent introduction of CIONM and its limited diffusion, a standardization of waveform interpretation is still necessary, despite numerous studies attempting to identify the correlation between neurophysiopathological data of nerve stimulation with the clinical data of cordal functionality. In 2018, Schneider et al., on the basis of the experiences of the working group and of the different publications previously reported, tried to formulate state-of-the art guidelines, defining reference parameters. From an empirical parallelism with the monitoring systems of other peripheral nerves, a signal with an initial amplitude equal to or greater than 500 µV was defined as adequate. Moreover, as reported by the INMSG, intraoperative LOS (Loss of signal) is defined as the loss of a recognizable EMG signal with curve amplitude below the 100 µV threshold (5, 8). Potentially harmful adverse events are identified by quantitative amplitude and latency variations characterizing the reference signal. Variations of the EMG signal can be single (decrease of amplitude or increase of latency) or combined (decrease of amplitude and increase of latency). A joint reduction >50% in amplitude and an increase >10% in latency lasting 40–60 s are related to a high risk of vocal cord paralysis. The authors suggest that the final wave amplitude value >250 µV correlates with the highest PPV and NPV results (75–99%); a final EMG signal with an amplitude >250 µV, which represents at least 50% of the baseline value, is strongly indicative of normal postoperative cordal function (5, 8). In our personal experience we analyzed the plots of 193 nerves. During each monitoring, the correct positioning of the endotracheal tube, the standardized use of short-acting muscle relaxants, and the functional integrity of the circuit connections with the generator were peremptorily ensured. In 110 cases, the acquired EMG signals met the criteria established by INMSG guidelines, with an initial amplitude value of at least 500 µV. In the other 83 cases, however, we weren’t able to obtain such an initial amplitude value, despite having ensured the correct positioning of endotracheal tube, the absence of neuromuscular blocking agents, and the functionality of the monitoring system. Nevertheless, we decided to perform intraoperative neuromonitoring in these cases, as the detected EMG waveforms were well recognizable and stable. Furthermore, in four cases the EMG signals showed an amplitude <100 µV since probe positioning and for the entire duration of the monitoring, despite a physiological cordal motility both preoperatively and postoperatively. The data analysis showed that an increase of VCP risk was associated with the progressive amplitude reduction during intervention in both groups (Group 1: initial stimulation amplitude ≥500 µV; Group 2: initial stimulation amplitude between 100 and 500 µV), corresponding to a mean increase of 5% of the relative risk of VCP per each percentage value of amplitude decrease. Furthermore, it allowed us to determine two cut-off values for amplitude reduction with optimal sensitivity and specificity: −77% in Group 1 and −15% in Group 2. The comprehensive evaluation of the EMG waveforms led to several questions, which still need to be answered:

- How should the surgeon approach a situation in which the initial response to vagal stimulation has an amplitude lower than 500 µV?
- Would it be plausible to perform continuous neuromonitoring in such situations?
- Should the resulting EMG waveforms be considered reliable, and should the surgeon trust them to perform intraoperative corrective maneuvers when the amplitude decreases?
- Can a statistical analysis be used to define specific threshold values for the neuromonitoring when the initial response has an amplitude lower than 500 µV?
- How to approach those cases in which the initial response has a well recognizable waveform with an amplitude lower than 100 µV, which is considered to be LOS as per INMSG guidelines?

Considering intra and inter-subject variability of nerve transmission, the use of an amplitude cut-off value ≥500 µV could be too restrictive. In our experience, waveforms with baseline amplitude <500 µV may be considered equally adequate, as shown in overlapping results in comparison groups. Setting the alarm for a reduction of 77% in patients with initial amplitude ≥500 µV and of 15% for those <500 µV could make monitoring safe and an effective aid for surgeon also in these cases.

## Conclusions

Intraoperative neuromonitoring represents a safe and effective method for leading the surgeon during dissection and identification of recurrent nerve; it is universally accepted as a method capable of intraoperatively assessing the nerve functional integrity and preventing bilateral cordal paralysis in total thyroidectomies.

According to a recent multicentric retrospective clinical trial, continuous nerve monitoring compared to intermittent one shows a better predictivity of vocal cord paralysis (15). While the costs of the procedure need to be considered, it is clear how the cost/effectiveness leads to the use of neuromonitoring, especially in selected cases.

Therefore, the surgeon must have clear elements to interpret correctly EMG plots and to use CIONM as a real aid in surgical practice. The literature shows unique data interpretations when the nerve responses have an initial amplitude ≥500 µV.

Our experience showed that there are cases in which initial amplitude is lower than that considered as adequate by current literature but with well recognizable and stable EMG waveforms.

How those cases should be approached and what should the surgeon’s attitude be are a matter of discussion; further studies on larger populations are required to address these issues.

## Data Availability Statement

The raw data supporting the conclusions of this article will be made available by the authors, without undue reservation.

## Ethics Statement

Ethical approval was not provided for this study on human participants because, based on the characteristics of the study, the Medical Management of the Hospital of Pordenone did not consider that the study needed approval. The patients/participants provided their written informed consent to participate in this study.

## Author Contributions

All authors listed have made a substantial, direct, and intellectual contribution to the work, and approved it for publication.

## Funding

The funding for the publication comes from SEDA-SPA.

## Conflict of Interest

The authors declare that the research was conducted in the absence of any commercial or financial relationships that could be construed as a potential conflict of interest.

## Publisher’s Note

All claims expressed in this article are solely those of the authors and do not necessarily represent those of their affiliated organizations, or those of the publisher, the editors and the reviewers. Any product that may be evaluated in this article, or claim that may be made by its manufacturer, is not guaranteed or endorsed by the publisher.
